# Self-rated health habits, mental health, emotional intelligence, and impulsivity across the Baltic States

**DOI:** 10.3389/fpubh.2025.1522918

**Published:** 2025-05-13

**Authors:** Albertas Skurvydas, Dovile Valanciene, Ausra Lisinskiene, Ruta Dadeliene, Asta Sarkauskiene, Andra Fernāte, Zermena Vazne, Juris Grants, Andre Koka, Daiva Majauskiene

**Affiliations:** ^1^Department of Rehabilitation, Physical and Sports Medicine, Institute of Health Sciences, Faculty of Medicine, Vilnius University, Vilnius, Lithuania; ^2^Education Academy, Vytautas Magnus University, Kaunas, Lithuania; ^3^Faculty of Law, Vilnius University, Vilnius, Lithuania; ^4^Institute of Education Studies, Education Academy, Vytautas Magnus University, K. Kaunas, Lithuania; ^5^Department of Sports, Recreation and Tourism, Klaipėda University, Klaipeda, Lithuania; ^6^Riga Stradins University, Latvian Academy of Sport Education, Riga, Latvia; ^7^Faculty of Exercise and Sports Sciences, Institute of Sport Pedagogy, University of Tartu, Tartu, Estonia

**Keywords:** physical activity, obesity, subjective health, perceived stress, healthy eating, Estonia, Latvia, Lithuania

## Abstract

**Introduction:**

The study aims to compare physical activity, sleep, body mass index (BMI), subjective health, stress, depression, impulsivity, and emotional intelligence across the Baltic countries while identifying key health determinants.

**Methods:**

We interviewed men and women (18–64 years) in Estonia (*n* = 1,503) Latvia (*n* = 1,563) and Lithuania (*n* = 2,358) via an online survey.

**Results:**

Obesity is most common among individuals in EE, both males and females, while the highest rates of overweight are observed in EE females and LT males. Sleep duration is comparable across the different countries, with women generally sleeping longer than men (*p* < 0.05). Sedentary behavior is shortest among LT females and LV males, although in all countries, females tend to sit more than males (*p* < 0.05). Moderate physical activity (MPA) levels are similar among males, but they are highest among EE females. In contrast, vigorous physical activity (VPA) is most prevalent in LV males and females. Emotional intelligence (EI) is lowest among LV females and EE males, while females consistently exhibit higher EI than males across all countries. Lithuanians frequently rate their health as “excellent” and show the lowest levels of impulsivity. Additionally, depression and stress are lowest in EE females and LV males. Estonians are more likely to eat breakfast regularly and tend to overeat less than their counterparts in LV and LT.

**Conclusion:**

Our research clearly shows that perceived health among the Baltic countries is significantly inversely related to age, perceived stress, depression, and Body Mass Index (BMI). In contrast, we found positive associations between vigorous physical activity (VPA) and emotional intelligence (EI).

## 1 Introduction

Numerous research studies show that physical activity is effective in combating many chronic diseases (1, 2) and improving well-being and mental health (3–8). It was found that the health benefits of physical activity depend on people’s age, gender, health, and body mass index (2, 4, 5). Moreover, it was clearly proven that independent of physical activity, total sitting time is associated with greater risk for several major chronic disease outcomes (9, 10). It was found that the mortality rate caused by all illnesses increase also due to too low and too high body weight (11). According to the European Association for the Study of Obesity Physical Activity Working Group recommendations, obesity can be regulated by emphasizing physical activity (especially aerobic) and healthy nutrition (12, 13). Moreover, sleep (14) and sedentary behavior (15) have been independently associated with body weight regulation. Evidence from epidemiological studies indicates that depression and obesity have a strong bidirectional relationship, i.e., BMI increases the risk for developing depression, and vice versa, individuals with depression have an increased risk of high BMI (16).

Though research demonstrates that EI does not depend on gender, obese surveyed individuals poorly managed their emotions compared to those whose body weight was normal (17). Individuals with obesity class III were characterized by a reduced trait of emotional intelligence and happiness and a higher tendency to use emotion suppression compared to normal weight individuals (18). All individuals with obesity also showed higher levels of depression. The research results show that depression has inverse correlation with EI (19), and the meta-analysis revealed a small but significant relationship between EI and sports performance (20). Physical activity is known to reduce depression (8). Meta-analysis showed a significant association between stress and BMI, waist circumference and serum triglyceride level (21). Besides, chronic stress and cortisol concentration correlate significantly positively with BMI (22). Obesity and mood disorders are two of the most serious health issues of modern times. These health conditions are often linked with obesity acting both as a cause and consequence of anxiety and depression (23, 24). Konttinen et al. has proven the hypothesis declaring that emotional eating is one behavioral mechanism between depression and development of obesity and abdominal obesity (23). Moreover, adults with a combination of shorter night sleep duration and higher emotional eating may be particularly vulnerable to weight gain.

Despite previous research, there are still few studies comparing cross-country differences in physical activity (PA), sleep, body mass index (BMI), healthy eating, emotional intelligence (EI), perceived stress, overall health, depression, and impulsivity. Additionally, there is a notable gap in research that examines key health determinants across countries. This includes not only physical activity, sleep, sedentary behavior, eating habits, BMI, age, and gender, but also mental health factors such as depression, impulsivity, and perceived stress, along with emotional intelligence.

Besides, physical activity in different countries usually cannot be compared because of different methods being employed (25).The aim of our research was to compare various aspects of physical activity (PA)—including sedentary behavior (SB), light intensity PA (LPA), moderate intensity PA (MPA), and vigorous PA (VPA)—as well as sleep patterns, body mass index (BMI) categories (<18.5, 18.5–24.9, 25–29.9, and ≥30 kg/m^2^), subjective health, perceived stress, depression, impulsivity, emotional intelligence (EI), and healthy eating indicators such as overeating and breakfast consumption among men and women aged 18 to 64 years in Estonia (EE), Latvia (LV), and Lithuania (LT). Additionally, we sought to identify which of the examined factors—gender, age, sleep duration, sedentary behavior, moderate physical activity, vigorous physical activity, BMI, depression, impulsivity, emotional intelligence, overeating, and breakfast consumption—had the most significant impact on subjective health in individuals from each Baltic country. All research cases conducted in various Baltic countries utilized the same methods.

## 2 Methods

### 2.1 Participants and procedures

We investigated and compared large voluntary samples of males and females (age range 18–64 years) in Estonia (*n* = 1,503), Latvia (*n* = 1,563) and Lithuania (*n* = 2,358) on self-rated health habits, mental health, EI, and impulsivity. We randomly selected Lithuanian, Estonian, and Latvian participants aged 18–64 years in good health to ensure a representative sample from the Baltic region. The optimal representative sample size needed to be at least 1,000 participants per country. However, we selected a larger sample to improve the accuracy and reliability of our conclusions. Participants completed the survey from 2019 October to June 2020. Participation was anonymous, and data collection and handling were confidential. We used an online survey to collect information through https://docs.google.com/forms/. All participants completed the online questionnaires in full or did not submit their form. We recruited participants through social media (i.e., Facebook). The research teams also used contacts to ask for help in publicizing our survey. The study was conducted according to the guidelines of the Declaration of Helsinki (26) and National guidelines for biomedical and health research involving human participants (27).

### 2.2 Measurements

#### 2.2.1 Demographics

We asked participants to self-report their country of residence, age, education, body mass index (BMI), athletic status (i.e., professional athlete or not) and whether they performed physical exercises independently or in sports and health centers.

#### 2.2.2 Danish physical activity questionnaire (DPAQ)

The DPAQ was adapted from the International Physical Activity Questionnaire and differs from it by referring to PA in the past 24 h (for 7 consecutive days) instead of the past 7 days. The selected activities were listed on the PA scale at nine levels of physical exertion in metabolic equivalents (METs), ranging from sleep or inactivity (0.9 MET) to highly strenuous activities (> 6 METs). Each level (A = 0.9 MET, B = 1.0 MET, C = 1.5 METs, D = 2.0 METs, E = 3.0 METs, *F* = 4.0 METs, G = 5.0 METs, H = 6.0 METs, and I > 6 METs) was described using examples of specific activities of a particular MET level and a small drawing. The PA scale was constructed so that the number of minutes (15, 30, or 45) and hours (1–10) spent at each MET activity level on an average 24-h weekday could be filled out. This allowed for a calculation of the total MET time, representing 24 h of sleep, work, and leisure time on an average weekday (28). We calculated how much energy (in METs) was consumed per day during sleep, sedentary behavior (SB), light intensity PA (LPA), moderate-intensity PA (MPA), and vigorous-intensity PA (VPA).

#### 2.2.3 Subjective health assessment

A four-point scale was used for this: poor health (1 point); satisfactory (2 points), good (3 points); excellent (4 points).

#### 2.2.4 Emotional intelligence (EI)

EI was assessed using the Schutte self-report emotional intelligence test (SSREIT) (29). The SSREIT is a 33-item questionnaire divided into four subscales: perception of emotion assessed by 10 items, managing own emotions assessed by 9 items, managing others’ emotions assessed by 8 items, and utilization of emotions assessed by 5 items. The items are answered on a five-point scale ranging from 1 (strongly disagree) to 5 (strongly agree). Total scores range from 33 to 165, with the higher scores indicating greater ability in EI.

#### 2.2.5 Perceived stress and depression assessment

The 10-item perceived stress scale (PSS-10) was used to measure participants’ stress levels (30). In the PSS-10, participants were asked to answer 10 questions about feelings and thoughts during the last month on Likert scale ranging from 0 (never) to 4 (very often), indicating how often they have felt or thought in a certain way within the past month. Scores range from 0 to 4, higher scores indicate higher levels of perceived stress. Depression was assessed according to a four-point visual scale, increasing from lower to higher depression.

#### 2.2.6 Assessment of impulsivity

Impulsivity was assessed using the Barratt Impulsiveness Scale Version 11 (BIS-11) (31). This scale is a 30-item questionnaire divided into three subscales: attentional impulsiveness assessed using eight items, motor impulsiveness assessed using 11 items, and non-planning impulsiveness assessed using 11 items. The items are answered on a 4-point scale ranging from 1 (rarely/never) to 4 (almost always/always). Total scores range from 30 to 120, with higher scores representing higher impulsivity.

#### 2.2.7 Eating breakfast and overeating

Eating breakfast and overeating were measured on a 3-pont (1—not, 2—sometimes, 3—yes).

### 2.3 Statistical analysis

We employed various statistical methods and analyses for our study. Initially, we confirmed that all interval data followed a normal distribution using the Kolmogorov–Smirnov test. We conducted two-way analyses of variance (ANOVA) to evaluate the effects of two independent variables: country (EE, LV, LT) and gender (male and female), on the dependent variables, which included age, body mass index (BMI), impulsivity, emotional intelligence (EI), sleep, sedentary behavior (SB), light physical activity (LPA), moderate physical activity (MPA), and vigorous physical activity (VPA). We defined statistical significance as *p* < 0.05 for all tests. Besides, we also calculated the effect size using eta squared (η^2^). If we identified significant effects, we utilized Tukey’s *post hoc* adjustment to account for multiple comparisons. To investigate the effects of gender, age, sleep duration, sedentary behavior (SB), moderate physical activity (MPA), vigorous physical activity (VPA), body mass index (BMI), depression, impulsivity, emotional intelligence, impulsivity, overeating, and breakfast consumption on subjective health, we conducted a linear regression analysis using standardized beta coefficients. This analysis was performed separately for Estonians (Model 1), Latvians (Model 2), and Lithuanians (Model 3). We aimed to examine the relationship between specific Baltic countries and various factors, including education, physical activity characteristics, body mass index (BMI) structure, health, depression, stress, breakfast habits, and overeating. To do this, we calculated the chi-square (χ^2^) statistic and its *p*-value separately for men and women. The statistical analyses were conducted using IBM SPSS Statistics software (version 22; IBM SPSS, Armonk, NY, USA). We categorized the participants based on their BMI into four groups: under 18.5 kg/m^2^; 18.5 to 24.9 kg/m^2^; 25 to 29.9 kg/m^2^; and 30 kg/m^2^ and above, also separated by gender.

## 3 Results

### 3.1 Education and characteristics of physical activity of the surveyed

Women with higher university-level education in Estonia (EE), Latvia (LV), and Lithuania (LT) represented 80.4, 63.9, and 72.6%, respectively (with a significant country effect: *p* < 0.001). In contrast, the percentages for men in these countries were 73.2, 39.2, and 71.3%, also showing a significant country effect (*p* < 0.001) (see Table 1). In terms of physical activity, the percentage of females who reported not exercising at all in EE, LV, and LT was 37.5, 32.4, and 39.3%, respectively (country effect: *p* < 0.001). For males, the figures were lower, at 23.6, 16.2, and 26.2% (country effect: *p* < 0.001) (see Table 2).

**Table 1:** Education of the surveyed.

Education		Female			Male		
		EST	LAT	LT	EST	LAT	LT
Not finished secondary school	Count	17_a_	17_a_	24_a_	4_a, b_	20_b_	7_a_
	% within education	29.3%	29.3%	41.4%	12.9%	64.5%	22.6%
	% within Country	1.3%	1.7%	1.3%	2.1%	3.4%	1.3%
Higher not university	Count	114_a_	42_b_	161_a_	18_a_	45_a_	41_a_
	% within education	36%	13.2%	50.8%	17.3%	43.3%	39.4%
	% within Country	8.6%	4.3%	8.8%	9.5%	7.7%	7.9%
Professional	Count	62_a_	89_b_	116_a_	14_a_	90_b_	35_a_
	% within education	23.2%	33.3%	43.4%	10.1%	64.7%	25.2%
	% within Country	4.7%	9.1%	6.3%	7.4%	15.4%	6.7%
Secondary	Count	66_a_	205_b_	203_c_	15_a_	201_b_	67_a_
	% within education	13.9%	43.2%	42.8%	5.3%	71%	23.7%
	% within Country	5%	20.9%	11.1%	7.9%	34.3%	12.8%
University	Count	1061_a_	626_b_	1333_c_	139_a_	230_b_	372_a_
	% within education	35.1%	20.7%	44.1%	18.8%	31%	50.2%
	% within Country	80.4%	63.9%	72.6%	73.2%	39.2%	71.3%

When the letters do not coincide, then *p* < 0.05.

**Table 2:** Characteristics of physical activity of the surveyed.

Do you play sports?		Female			Total	Male			Total
		EST	LAT	EST		LAT	EST	LAT	
I do not play any sports	Count	495_a_	317_b_	722_a_	1,534	50_a_	95_b_	137_a_	282
	% within Play Sports	32.3%	20.7%	47.1%	100%	17.7%	33.7%	48.6%	100%
	% within Country	37.5%	32.4%	39.3%	37.1%	26.3%	16.2%	26.2%	21.7%
I do sports at health club	Count	201_a_	90_b_	162_b_	453	36_a_	81_a_	36_b_	153
	% within Play Sports	44.4%	19.9%	35.8%	100%	23.5%	52.9%	23.5%	100%
	% within Country	15.2%	9.2%	8.8%	11%	18.9%	13.8%	6.9%	11.8%
I do sports individually by my self	Count	607_a_	511_b_	909_a, b_	2027	95_a_	347_b_	303_a, b_	745
	% within Play Sports	29.9%	25.2%	44.8%	100%	12.8%	46.6%	40.7%	100%
	% within Country	46%	52.2%	49.5%	49%	50%	59.2%	58%	57.4%
I do sports professionally	Count	17_a_	61_b_	44_c_	122	9_a_	63_b_	46_a, b_	118
	% within Play Sports	13.9%	50%	36.1%	100%	7.6%	53.4%	39%	100%
	% within Country	1.3%	6.2%	2.4%	2.9%	4,7%	10.8%	8.8%	9.1%

When the letters do not coincide, then *p* < 0.05.

### 3.2 Body mass index

**Figure 1:**
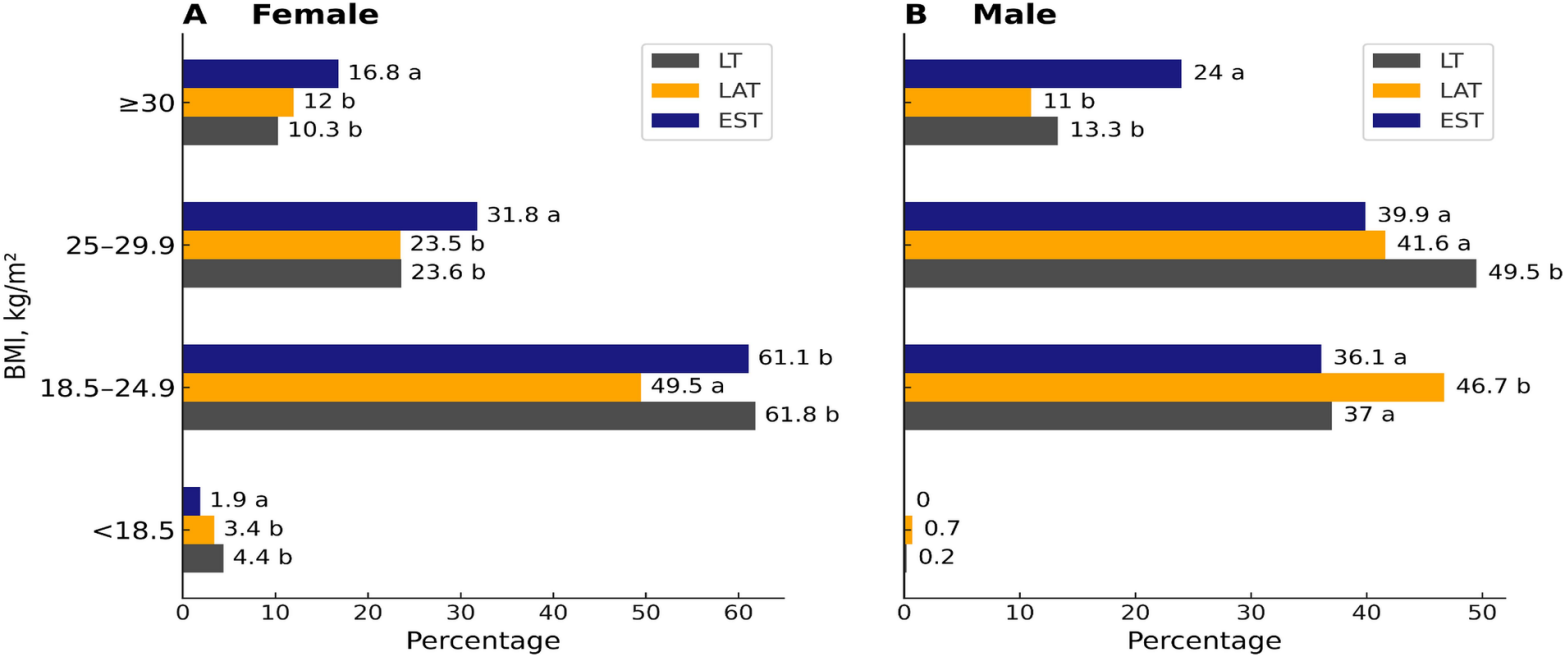
Percentage of BMI structure in females and males of different countries. When the letters do not coincide, then *p* < 0.05. Pearson Chi-Square in females: 84.9; *p* < 0.001; Pearson Chi-Square in males—32.9; *p* < 0.001.

The Body Mass Index (BMI) of males and females in EE was higher than that of those in LV and LT, with a statistically significant difference (*p* < 0.05). However, there was no significant difference in BMI between LV and LT for both genders (*p* > 0.05) (see Table 3). When comparing the BMI of males and females across all countries, males consistently had higher BMI values than females (*p* < 0.05). Additionally, a greater number of males and females from EE had a BMI greater than 30 kg/m^2^ compared to those from LV and LT (*p* < 0.05), while no significant difference was observed between LV and LT (*p* > 0.05) (see Figure 1). Moreover, there were more overweight females in EE than in LV and LT (*p* < 0.05). Conversely, LT had a higher number of overweight males compared to those in EE and LV (*p* < 0.05). Overall, overweight males outnumbered females in all countries (*p* < 0.05). Interestingly, there were fewer females in Estonia with a normal BMI compared to those in LV and LT (*p* < 0.05), while the difference between LV and LT females was not statistically significant (*p* > 0.05). Among males, the highest proportion with a normal BMI was found in LV (*p* < 0.05), whereas there was no significant difference in the percentage of males with a normal BMI between EE and LT (*p* > 0.05). In terms of insufficient BMI, the lowest figure was observed among females in EE (*p* < 0.05), while no significant differences were detected among males from EE, LV, and LT (*p* > 0.05).

**Table 3:** Descriptive characteristics (mean ± sd) of females and males in different countries.

Gender		Number of participants	Age, years	SD	Weight, kg	SD	Height, m	SD	BMI, kg/m^2^	SD
Female	EST	1,313	47.9_a_	13.1	72.3_a_	14.9	1.66_a_	8.3	25.9_a_	5.2
	LAT	978	37.5_b_	16	69_b_	13.8	1.70_b_	57.8	24.4_b_	4.8
	LT	1837	398_c_	12.3	68.9_b_	14.4	1.68_b_	6.8	24.2_b_	4.9
Male	EST	190	45.7_a_	14	88.8_a_	15.8	1.81_a_	7	27_a_	4.6
	LAT	585	33.2_b_	14.1	84.9_b_	13.1	1.84_b_	63.8	25.6_b_	3.5
	LT	521	39.8_c_	12.8	87.3_c_	13.2	1.85_b_	78.9	26.2_b_	3.6
	Country effect:									
	*p*-value		0.000						0.000	
	η^2^		0.057						0.008	
	Gender effect:									
	*p*-value		0.000						0.000	
	η^2^		0.014						0.014	
	Interaction effect: *p*-value		0.000						0.026	

When the letters do not coincide, then *p* < 0.05.

### 3.3 Breakfast and overeating

The research results show that both females and males in EE more often eat breakfast and overeat less than those in LV and LT (Table 4).

**Table 4:** Eating breakfast and overeating (in per-cent) in females and males of Estonia (EST), Latvia (LAT) and Lithuania (LT).

	Gender	EST	LAT	LT	Effect of countries, *p*-value
Breakfast	Female	83.2_a_	73.7_b_	71.7_b_	<0.001
Breakfast	Male	80a	71_b_	75.9_b_	<0.001
Non-overeating	Female	31.7_a_	21.4_b_	20.5_b_	<0.001
Non-overeating	Male	30a	20.8_b_	21.1_b_	<0.001

When the letters do not coincide, then *p* < 0.05.

### 3.4 Sleep, SB, and PA

**Figure 2:**
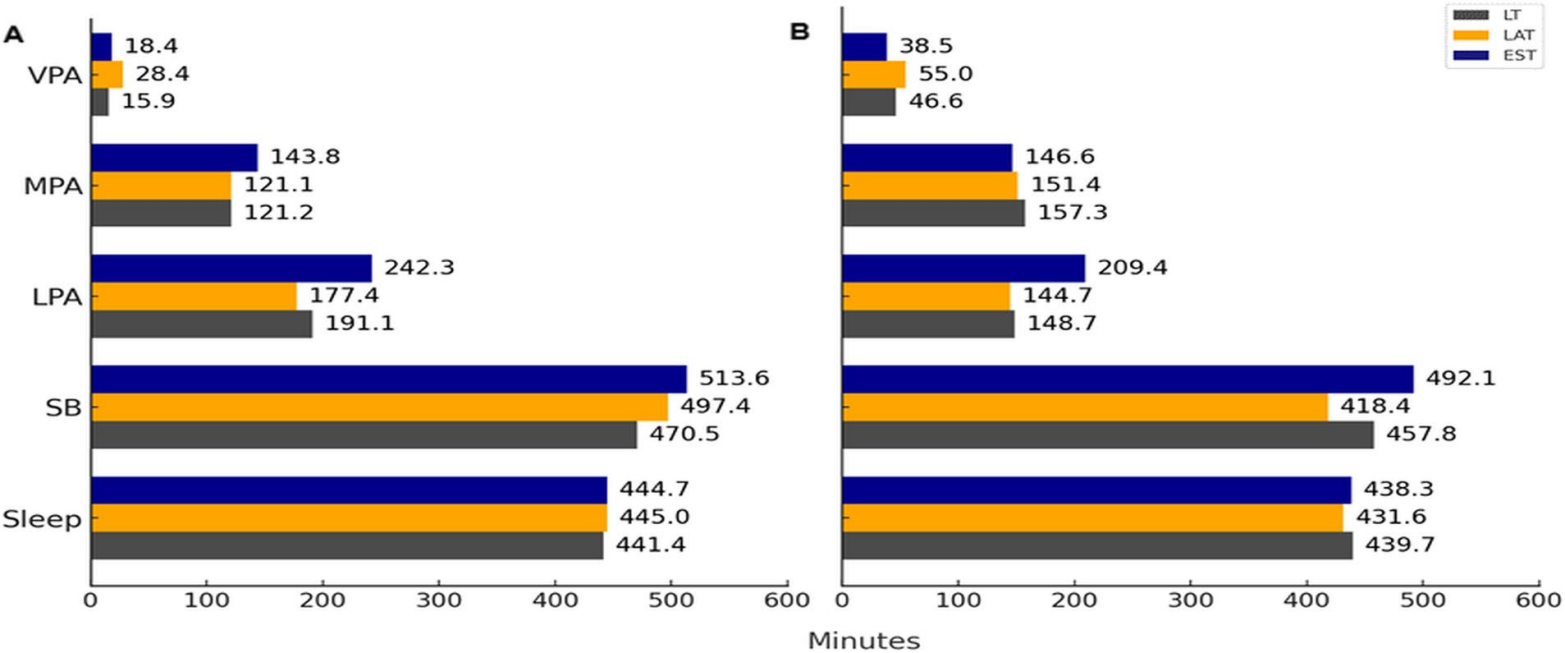
Sleep, sedentary behavior (SB), light intensity physical activity (LPA), moderate intensity physical activity (MPA), vigorous intensity physical activity (VPA) in males and females in different countries (mean ± SD). When the letters do not coincide, then *p* < 0.05.

A two-way ANOVA analysis revealed significant effects of country and gender on sleeping time, sedentary behavior (SB), light physical activity (LPA), moderate physical activity (MPA), and vigorous physical activity (VPA). The outcomes were as follows: for sleeping time for SB, country (*p* = 0.55; η^2^ < 0.0001), gender (*p* = 0.005; η^2^ = 0.001), and their interaction (*p* = 0.076); for SB, country (*p* < 0.001; η^2^ = 0.007), gender (*p* < 0.001; η^2^ = 0.004), and their interaction (*p* < 0.001) were all significant; for LPA, country (*p* < 0.001; η^2^ = 0.031) and gender (*p* < 0.001; η^2^ = 0.016) were significant, but the interaction was not (*p* = 0.45); for MPA, country (*p* = 0.007; η^2^ = 0.002), gender (*p* < 0.001; η^2^ = 0.004), and the interaction (*p* = 0.024) were significant; and for VPA, country (*p* < 0.001; η^2^ = 0.01), gender (*p* < 0.001; η^2^ = 0.044), and the interaction (*p* = 0.042) were significant (see Figure 2). The results indicated that females tended to sleep and sit longer, while their LPA was higher. In contrast, their MPA and VPA were shorter compared to males. VPA levels for both low-volume (LV) males and females were higher than those for LV and low training (LT) individuals (*p* < 0.05). Among EE females, both MPA and LPA were higher than those of LV and LT females (*p* < 0.05). However, MPA among males from different countries did not show significant differences (*p* > 0.05), while LPA for EE males was significantly higher than that of LV and LT males (*p* < 0.05). There were no significant differences in sleep duration for males or females across different countries (*p* > 0.05). SB for both EE males and females was longer than that for LV and LT individuals (*p* < 0.05). Notably, the shortest SB was observed in LT females and LV males (*p* < 0.05 when compared to individuals from other countries).

### 3.5 Health, depression, and perceived stress

**Figure 3:**
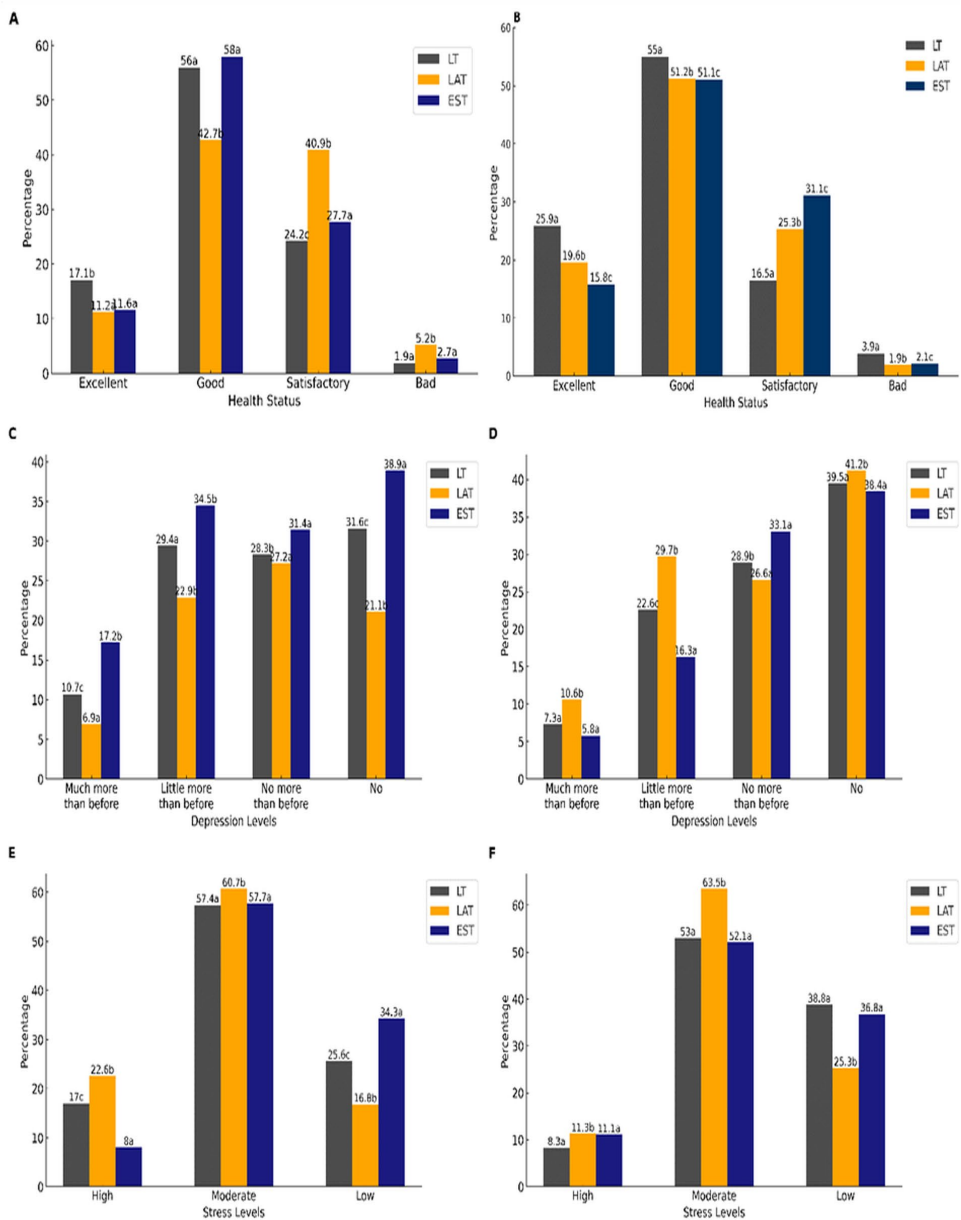
Percentage of structure health in female and male of different countries **(A,B)**. When the letters do not coincide, then *p* < 0.05. Pearson Chi-Square in females: 159.4; *p* < 0.001; Pearson Chi-Square in males—36.1; *p* < 0.001. Percentage of structure depression in females and males of different countries **(C,D)**. When the letters do not coincide, then *p* < 0.05. Pearson Chi-Square in females: 141.5; *p* < 0.001; Pearson Chi-Square in males—30.7; *p* < 0.001. Percentage of structure perceived stress in females and males of different countries **(E,F)**. When the letters do not coincide, then *p* < 0.05. Pearson Chi-Square in females: 149.2; *p* < 0.001; Pearson Chi-Square in males—26.1; *p* < 0.001.

In LT, both males and females rated their health as “excellent” significantly more often than those from other countries (*p* < 0.05). However, there was no notable difference between EE and LV in this regard (see Figure 3A). Across all countries surveyed, more males than females assessed their health as “excellent” (*p* < 0.05). In LV, females predominantly rated their health as “bad” or “satisfactory,” while this trend was not observed among females in EE or LT (*p* < 0.05). Additionally, more LV males rated their health as “bad,” but again, this was not the case for males in EE or LT (*p* < 0.05). When it comes to feelings of depression, a greater number of EE females reported not experiencing any depressive feelings compared to those from LT and LT (*p* < 0.05), with a significant difference also observed between LT and LV (*p* < 0.05) (see Figure 3B). Likewise, more males from both LT and EE reported not feeling any depression compared to their LV counterparts (*p* < 0.05). Conversely, a higher number of LV males and females indicated that their levels of depression had increased significantly (*p* < 0.05). Stress levels varied among the countries as well. Most LT females reported feeling “high” stress, which was significantly higher than their counterparts in LT and EE (*p* < 0.05). Conversely, EE females reported the least amount of stress compared to both LT and LT females (*p* < 0.05) (see Figure 3C). Among the males, the least number of LT individuals reported feeling “low” stress, in comparison to their LT and EE peers (*p* < 0.05), although no significant difference was found between the stress levels of LT and EE males (*p* > 0.05).

### 3.6 Emotional intelligence and impulsivity

**Figure 4:**
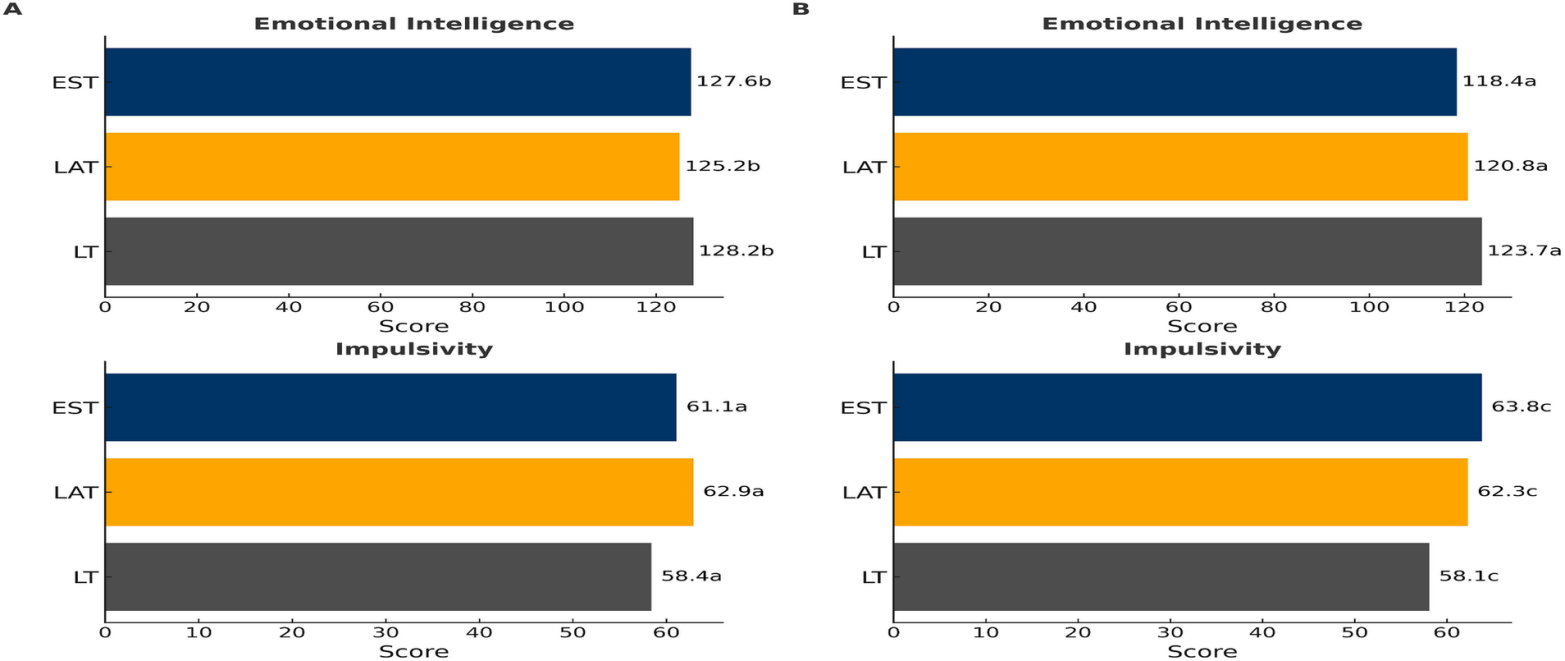
Emotional intelligence (EI). **(A)** and Impulsivity **(B)** in males and females in different countries (mean ± SD). When the letters do not coincide, then *p* < 0.05. Country effect: *p* = 0.000; gender effect: *p* = 0.01; interaction gender × country effect: *p* = 0.004.

Emotional Intelligence (EI) was found to be influenced by both country (*p* < 0.001; η^2^ = 0.002) and gender (*p* < 0.001; η^2^ = 0.024), with a significant interaction effect between gender and country (*p* = 0.002) (see Figure 4). The effects of gender (*p* = 0.01; η^2^ = 0.002) and country (*p* < 0.001; η^2^ = 0.027) were significant, with an interaction between gender and country (*p* = 0.0044). Among the participants, LT males exhibited the highest EI (*p* < 0.05 when compared to EE), while EE males had the lowest EI (*p* < 0.05 when compared to LT). Additionally, LV females showed significantly lower EI compared to both LT and EE females (*p* < 0.05). Studies show that impulsivity significantly depends on country (*p* < 0.0001; η^2^ = 0.027) and gender (p = 0.01; η^2^ = 0.002), with a significant interaction between gender and country (*p* = 0.0044). In terms of impulsivity, LT males and females demonstrated the lowest levels when compared to both EE and LV participants (*p* < 0.05) (refer to Figure 4). On the other hand, EE females displayed the highest levels of impulsivity (*p* < 0.05 when compared to LT and LV females). For males, the highest impulsivity was observed in both LV and EE.

### 3.7 Predictors of subjective health

**Figure 5:**
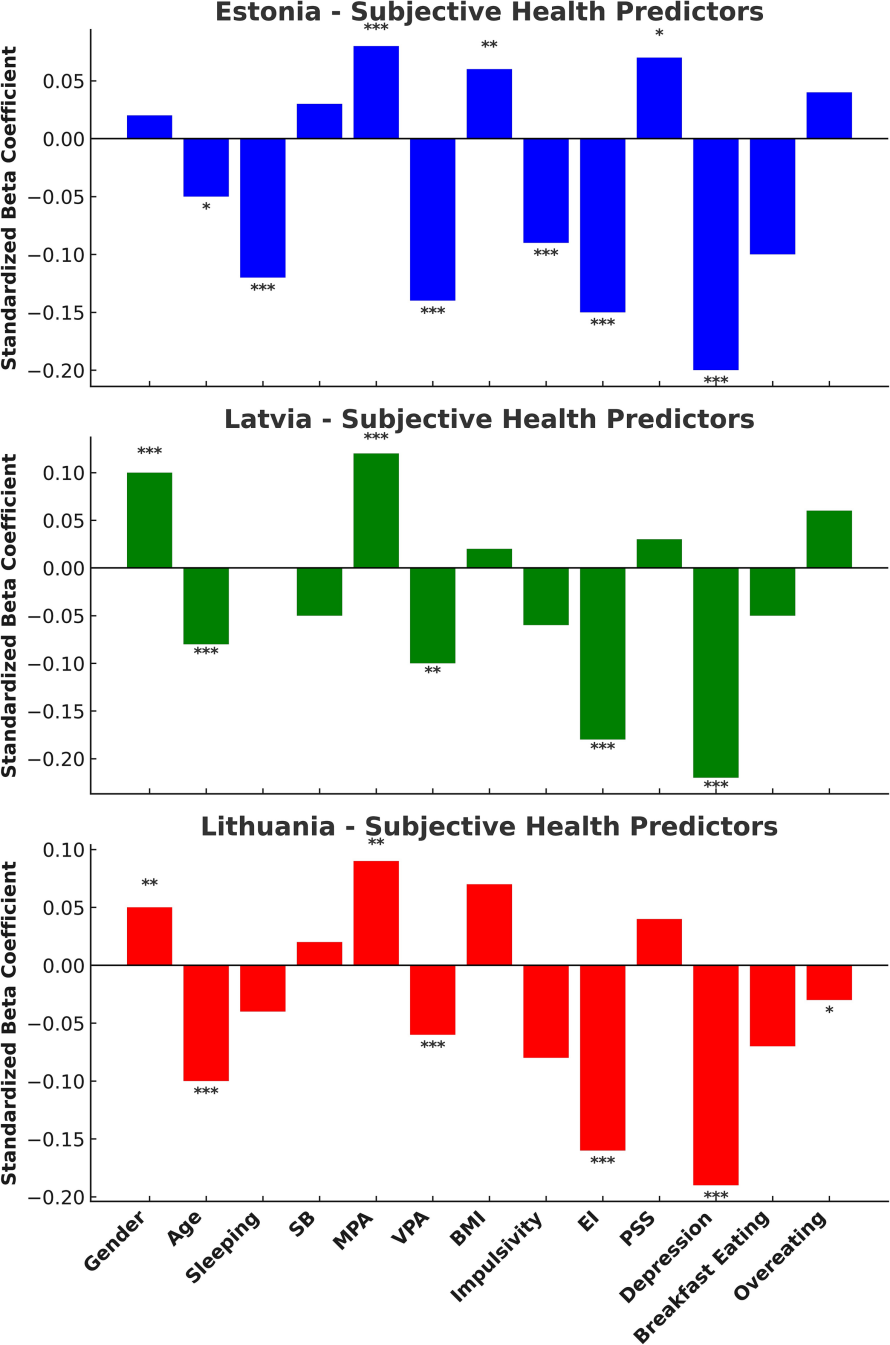
Standardized beta coefficients for predictors of subjective health in Estonia, Latvia, and Lithuania. Significant predictors are marked as *p* < 0.05 (*), *p* < 0.01 (**), *p* < 0.001 (**).

Subjective health across all Baltic countries is significantly and inversely related to age, perceived stress (PSS), depression (*p* < 0.001), and BMI (*p* < 0.01 − < 0.001) (Figure 5). Positive associations are observed with vigorous physical activity (VPA) (*p* < 0.01 − < 0.001) and emotional intelligence (EI) (*p* < 0.05 − < 0.01). Notable gender differences are found in LV and LT, where men report better subjective health compared to women (*p* < 0.01). Additionally, in EE, the impact of eating breakfast on health has been found to be significant. Interestingly, neither sleep nor sedentary behavior (SB) had a significant effect on subjective health across the Baltic countries.

## 4 Discussion

To our knowledge, this research is the first to compare various aspects of physical activity (PA) including sedentary behavior (SB), light-intensity PA (LPA), moderate-intensity PA (MPA), and vigorous PA (VPA). Additionally, it examines sleep patterns, body mass index (BMI) categories (<18.5, 18.5–24.9, 25–29.9, and ≥30 kg/m^2^), as well as subjective health, perceived stress, depression, impulsivity, emotional intelligence (EI), and healthy eating habits (including breakfast consumption and overeating) among adult males and females across the Baltic region. Our research, which employed regression analysis (see Figure 5), shows that perceived health in the Baltic countries is significantly and inversely related to factors such as age, perceived stress, depression, and Body Mass Index (BMI). In contrast, we found positive associations with vigorous physical activity (VPA) and emotional intelligence (EI). Thus, despite variations in specific indicators among the Baltic countries, the key determinants of health remain consistent: older age is associated with poorer health; higher stress and depression levels correlate with worse health outcomes; a higher BMI is linked to poorer health; while greater vigorous physical activity (VPA) and higher emotional intelligence (EI) are associated with better health.

Our findings support previous research indicating that vigorous physical activity (VPA), body mass index (BMI), perceived stress (PSS), depression, and emotional intelligence (EI) significantly affect health. Studies confirm that physical activity reduces the risk of chronic diseases (1–8), while BMI has an influence on mortality and mental health (11, 16). Obesity is associated with difficulties in emotional regulation (17, 18), depression (19), and chronic stress (21, 22). Additionally, factors such as sedentary behavior (9, 10), sleep (14), and emotional eating (23) play important roles in weight management. It is notable that sleep duration, sedentary behavior (SB), and impulsivity were not significant health determinants for individuals in any of the Baltic countries. Additionally, breakfast consumption and overeating have a weaker impact on health compared to vigorous physical activity (VPA), body mass index (BMI), perceived stress scale (PSS), depression, and emotional intelligence (EI). Together, these factors underscore the complex relationship between physical, psychological, and emotional health.

Obesity and low physical activity are often interrelated — low physical activity contributes to obesity, while obesity reduces motivation for physical activity (32) and leads to more frequent overeating due to difficulties in resisting food cravings (33). Numerous studies indicate that BMI is influenced by physical activity (PA) and, more significantly, by eating habits (13, 15).

We found out that there were most of obese males and females in EST, even though they eat breakfast more often and overeat more seldom than males and females of other countries. However, SB is the highest in EST females. There are more males with normal body weigh in Latvia because they sit for long hours less and their VPA is higher. Nevertheless, it remains unclear why there are most of obese males and females in EST, if their VPA does not differ from that of LT, MPA of males does not differ among the countries, and EE females’ MPA is even higher than that for other countries. Age may be a significant factor in this context, as life expectancy is higher in Estonia than in many other countries. As people age, their basic metabolism tends to slow down, which can lead to an increased risk of obesity (34).

Additionally, the impact of prolonged sedentary behavior on health may also play a role; however, our regression analysis did not find evidence to support that sedentary behavior has a direct effect on health.

Interesting to note, in all the surveyed countries there were more females who had normal BMI than males, despite females of all the countries had lower MPA and VPA, and their SB was higher. Besides, there is no significant difference between males and females (for all the surveyed countries) in terms of eating breakfast and overeating frequency.

Therefore, a question why females in all the countries are slimmer than men remain open. This coincides with conclusions of our earlier investigations pointing out that BMI of Lithuanian females was significantly lower than that of males (4, 5). One of the recent meta-analyses (13.2 million subjects) showed that even though females around the world are still those who are overweight, recently, males are getting closer to that indicator value, too (35). What is more, our research demonstrated that duration of sleep did not differ for the surveyed countries (even though females in all the surveyed countries sleep longer than males). Interesting to note that Kocevska et al., found out that women (≥41 years) reported sleeping shorter times than men, whereas by employing actigraphy they were estimated to sleep longer than men (36).

We discovered that EI of females in all the surveyed countries was higher than that of males, and this coincides with our earlier research conducted in Lithuanian population (4, 5, 37). This is also in line with a conclusion of the investigation carried out by Cabello et al. emphasizing that EI was greater in women than in men (38). However, Miguez-Torres et al., found out that EI does not depend on gender, and obese surveyed individuals demonstrated poorer management of their emotions rather than those with normal body weight (17). A similar conclusion was also made by Andrei et al. underlining that individuals with obesity class III were characterized by a reduced trait of emotional intelligence and happiness, and a higher tendency to use emotion suppression compared to normal weight individuals (18). Besides, Andrei et al. found out that all individuals with obesity also showed higher levels of depression (18). A similar conclusion was drawn by Luppino et al., declaring that depression inversely depends on EI (16). According to the data by Kopp and Jekauc (20) and Konttinen et al. (23), there is a significant correlation relationship between BMI and depression. The data of our research coincides with that of Lloyd et al. (19) revealing a significant relationship between EI and sports performance (20), and that exercise induced positive changes in quality of life but did not reduce depression. One of the recent meta-analyses showed a significant association between stress and BMI (21). Besides, other research studies demonstrated that chronic stress and cortisol concentration correlated significantly positively with BMI (22). Cortisol concentration showed a significant positive correlation with diabetes and obesity and remained a major predictor of coronary atherosclerosis (22). One limitation of our study was that we were unable to survey individuals of the same age from all three Baltic countries. Our findings indicated that participants from EE were older than those from LV and LT. Furthermore, we lack information on potential differences in dietary habits. Another limitation is the predominance of women in the samples from all three Baltic countries. Additionally, our recently published studies suggest that leisure-time physical activity has a significantly more positive impact on mental health than physical activity performed during the workday. Therefore, future research should take this aspect into account (39, 40).

## 5 Conclusion

Despite these limitations and variations in health-related and lifestyle indicators across countries, our research clearly demonstrates that perceived health in the Baltic countries is significantly and inversely associated with age, perceived stress, depression, and BMI, while showing positive associations with vigorous physical activity and emotional intelligence.

## Data Availability

The original contributions presented in the study are included in the article/supplementary material, further inquiries can be directed to the corresponding author.
